# Morphine Induces Albuminuria by Compromising Podocyte Integrity

**DOI:** 10.1371/journal.pone.0055748

**Published:** 2013-03-29

**Authors:** Xiqian Lan, Partab Rai, Nirupama Chandel, Kang Cheng, Rivka Lederman, Moin A. Saleem, Peter W. Mathieson, Mohammad Husain, John T. Crosson, Kalpna Gupta, Ashwani Malhotra, Pravin C. Singhal

**Affiliations:** 1 Renal Molecular Research Laboratoy, Feinstein Institute for Medical Research, Hofstra North Shore LIJ Medical School, Great Neck, New York, United States of America; 2 Academic Renal Unit, Southmead Hospital, Bristol, United Kingdom; 3 Department of Lab Medicine Pathology, Hennepin County Medical Center, University of Minnesota Medical School, Minneapolis, Minnesota, United States of America; 4 Division of Hematology, Oncology and Transplantation, Department of Medicine, University of Minnesota Medical School, Minneapolis, Minnesota, United States of America; Fondazione IRCCS Ospedale Maggiore Policlinico & Fondazione D'Amico per la Ricerca sulle Malattie Renali, Italy

## Abstract

Morphine has been reported to accelerate the progression of chronic kidney disease. However, whether morphine affects slit diaphragm (SD), the major constituent of glomerular filtration barrier, is still unclear. In the present study, we examined the effect of morphine on glomerular filtration barrier in general and podocyte integrity in particular. Mice were administered either normal saline or morphine for 72 h, then urine samples were collected and kidneys were subsequently isolated for immunohistochemical studies and Western blot. For *in vitro* studies, human podocytes were treated with morphine and then probed for the molecular markers of slit diaphragm. Morphine-receiving mice displayed a significant increase in albuminuria and showed effacement of podocyte foot processes. In both *in vivo* and *in vitro* studies, the expression of synaptopodin, a molecular marker for podocyte integrity, and the slit diaphragm constituting molecules (SDCM), such as nephrin, podocin, and CD2-associated protein (CD2AP), were decreased in morphine-treated podocytes. *In vitro* studies indicated that morphine modulated podocyte expression of SDCM through opiate mu (MOR) and kappa (KOR) receptors. Since morphine also enhanced podocyte oxidative stress, the latter seems to contribute to decreased SDCM expression. In addition, AKT, p38, and JNK pathways were involved in morphine-induced down regulation of SDCM in human podocytes. These findings demonstrate that morphine has the potential to alter the glomerular filtration barrier by compromising the integrity of podocytes.

## Introduction

Morphine, a metabolite of heroin, is the main stay of pain management after surgery, angina, myocardial infarction, and trauma [1]. However, heroin has also been one of the main drugs abused for addiction, and has significantly contributed to morbidity and mortality since the early 1990s in the United States [2], [3], [4], [5].

Morphine has been reported to exert a bimodal effect on the growth of kidney fibroblasts and glomerular mesangial cells [6], [7], [8], [9], [10]. Intravenous opiate addiction has also been considered a risk factor for the development of human immunodeficiency (HIV)-associated nephropathy [11], [12]. Johnson *et al* reported that morphine administration altered the microprojections on podocytes [13]. We also reported earlier that morphine promoted glomerular epithelial cell proliferation at lower concentration while triggered apoptosis at higher concentration [8]. We demonstrated that morphine-induced alteration in epithelial cell phenoytype was mediated by alteration of hemoxygenase (HO)-1 activity; however, in these studies, we did not study any loss of visceral epithelial cell (podocyte) markers in response to morphine [8]. Thus, the present study not only provides data on the effect of morphine on glomerular visceral epithelial cells (podocytes) *in vivo*, but also examines the effect of morphine on the markers of visceral epithelial cell integrity.

As the major constituent of glomerular filtration barrier (GFB), slit diaphragm (SD) plays an important role in the prevention of glomerular protein leakage both in physiological and in pathological states. SD consists of proteins specifically expressed by podocytes, such as nephrin, podocin, CD2-associated protein (CD2AP), and synaptopodin [14]. On that account, the podocyte is considered the key cell contributing to the development of albuminuric kidney diseases. Decrease of nephrin and podocin has been shown to be linked with albuminuria and progressive renal disease [15], [16]. Whether morphine impairs the SD constituting molecules (SDCM) has yet to be studied.

In the present study, we evaluated the effect of morphine on glomerular filtration barrier in general and podocyte integrity in particular. We showed that morphine-receiving mice not only developed albuminuria but also displayed attenuated expression of SDCM in human podocytes. We also demonstrated that morphine-induced loss of podocyte integrity through loss of slit diaphragm associated molecules is mediated by reactive oxygen species (ROS) generation. These findings provide a basis to support the hypothesis that morphine-induced direct impairment of podocytes contributes to kidney disease.

## Materials and Methods

### Reagents

Morphine pellets were obtained from National Institute on Drug Abuse (Bethesda, MD, USA). For *in vitro* study, morphine was dissolved in normal saline, stored at a concentration of 10^−1^ M, and used in concentrations of 10^−10^ to 10^−6^ M. [D-Pen^2,5^]-Enkephalin (DPDPE), [D-Ala^2^, N-Me-Phe^4^, Gly^5^-ol]-Enkephalin (DAMGO), 2-(3,4-Dichlorophenyl)-N-methyl-N-[(1S)-1-(2-isopropyl)-2-(1-(3-pyrrolinyl))ethyl] acetamide hydrochloride (LPK26) were obtained from Sigma Aldrich (St. Louis, MO). LY294002, SP600125, and SB203580 were purchased from Cell Signaling Technology (Danvers, MA).

### Animal experiments

All animal experiments complied with Institutional Animal Care and Use Committee (IACUC)-approved protocols. FVB/N mice (purchased from Jackson Laboratory, eight in each group) were administered either normal saline or morphine (by subcutaneous implantation of a 75-mg slow-release morphine pellet). After 72 h, spot urine samples were collected by giving gentle pressure over the urinary bladder. The mice were subsequently sacrificed and the kidneys were isolated for renal immunohistochemical and Western blotting studies. Albumin and creatinine in the urine were measured with Albuwell M Test Kit (Cat# 1011, Exocell, Philadelphia, PA) and Creatinine Companion (Cat# 1012, Exocell), respectively, following the instruction of the manufacturer.

### Culture of human podocytes

Human podocytes were cultured as previously reported [17]. Briefly, immortalized human podocytes proliferated in the growth medium containing RPMI 1640 supplemented with 10% fetal bovine serum, 1 X pecicillin-streptomycin, 1 mM L-glutamine, and 1 X insulin, transferrin, and selenium (ITS) (Invitrogen, Grand Island, NY) at permissive temperature (33°C). When the cells reached about 80% confluence, they were transferred to 37°C for differentiation in a medium without ITS for 7 days.

### RT-PCR

Total RNA was isolated from human podocytes using Trizol reagent (Invitrogen). Five micrograms of total RNA were reverse transcribed using the first-strand synthesis system (Invitrogen). PCR was performed by using Platinum PCR SuperMix High Fidelity (Invitrogen). Sequences of primers for human opiate receptor genes were: ACGTGCTTGTCATGTTCGGCATCGT (DOR-FW), ATGGTGAGCGTGAAGATGCTGGTGA (DOR-RV), GATACACAAAGATGAAGACAGCAACCAAC (KOR-FW), TCCCTGACTTTGGTGCCTCCAAGGACTATT (KOR-RV), GATCATGGCCCTCTACTCCA (MOR-FW), and GCATTTCGGGGAGTACGGAA (MOR-RV). GAPDH was used as internal control, and the primers were GGGAAGCTCACTGGCATGGCCTTCC (GAPDH-FW) and CATGTGGGCCATGAGGTCCACCAC (GAPDH-RV). Amplification was performed at 95°C for 5 min, followed by 30 cycles at 94°C for 1 min, 55°C for 30 s, 68°C for 1 min with a final extension cycle for 5 min at 68°C. DNA samples were visualized by 2% agarose gel electrophoresis.

### Immunofluorescent microscopy

Immunofluorescent microscopy was performed as previously reported [18]. Briefly, the kidneys were perfused in situ and then fixed with fresh 4% PFA and stored at −80°C. Subsequently, paraffin sections (4 μm) were prepared and de-paraffinized in xylene and re-hydrated through graded concentrations of alcohol. Epitope retrieval was carried out by heating the samples at 98°C for 2 h in Retrieveall-1 (Signet Laboratories, Inc.). Subsequently, cooled samples were permeabilized with 0.3% triton X-100 for 10 min, and were blocked with 2% BSA in 0.1% triton X-100 for 1 h at room temperature. Sections were then incubated with primary antibodies overnight at 4°C, followed by Alexa Fluor secondary antibodies (Invitrogen, 1∶800), donkey anti-rabbit IgG Alexa Fluor 488, donkey anti-goat lgG Alexa Fluor 594, goat anti-mouse IgG Alexa Fluo 488, or goat anti-rabbit IgG Alexa Fluo 568, for 1 hour at room temperature. Primary antibodies included rabbit anti-Mu/Kappa/Delta opiate receptors (Abcam, 1∶200), rabbit anti-synaptopodin (Santa Cruz, 1∶100), mouse anti-8-OHdG (Santa Cruz, 1∶100), and goat anti-nephrin (R&D systems, 1∶50). All antibodies were diluted in 0.1% Triton X-100, 2% BSA in PBS. Cells were then counterstained with DAPI to identify nuclei (Sigma-Aldrich). Morphological changes were visualized and captured with a ZEISS microscope (Carl Zeiss MicoImaging GmbH, Jena, Germany) equipped with a digital imaging system.

### Western blot analysis

Cells were rinsed twice with PBS and lysed by M-PER Protein Extraction Buffer (Pierce, Rockford, IL) containing 1× protease inhibitor cocktail (Roche Diagnostics, Indianapolis, IN). Proteins (20–30 μg) were separated by 10% SDS-polyacrylamide gel electrophoresis (PAGE) and then transferred to an Immuno-Blot polyvinylidene fluoride (PVDF) membrane (Bio-Rad, Hercules, CA). After blocking in PBS/Tween (0.1%) with 5% nonfat milk, the membrane was incubated with primary antibodies overnight at 4°C followed by horseradish peroxidase-conjugated secondary antibodies (Santa Cruz, 1∶3000) and then developed using Enhanced Chemiluminescent (ECL) solution (Pierce). Primary antibodies included rabbit anti-nephrin (Santa Cruz, 1∶1000), goat anti-synaptopodin (Santa Cruz, 1∶1000), mouse anti-CD2AP (Santa Cruz, 1∶1000), rabbit anti-podocin (Sigma, 1∶200), goat anti-actin (Santa Cruz, 1∶1000), rabbit anti-phospho-JNK (Cell Signaling, 1∶1000), rabbit anti-JNK (Cell Signaling, 1∶1000), rabbit anti-phospho-Akt (Cell Signaling, 1∶1000), rabbit anti-Akt (Cell Signaling, 1∶1000), rabbit anti-phospho-p38 (Cell Signaling, 1∶1000), and rabbit anti-p38 (Cell Signaling, 1∶1000). For data quantification, the films were scanned with a CanonScan 9950F scanner and the acquired images were then analyzed using the public domain NIH image program (developed at the U.S. National Institutes of Health and available on the internet at http://rsb.info.nih.gov/nih-image/).

### Intracellular ROS measurement

Human podocytes were differentiated in 96-well plates for 7 days as mentioned above, and were then cultured in serum free medium for 12 h. Subsequently, 10^−10^ to 10^−6^ M morphine was added. After incubation for another 12 h, intracellular ROS was determined by analyzing the fluorescence intensity of the intracellular fluoroprobe 2, 7-dichlorofluorescin (DCFH) (Molecular probe, Carlsbad, CA, USA), following the manufacturer's instructions.

### Electron Microscopic (EM) studies

Mice in groups of five were administered either normal saline or morphine. Kidneys were harvested and fixed in glutaraldehyde and embedded in EPON. Thin sections were cut and reviewed and examined under a transmission microscope. All the EM sections were reviewed in a blinded fashion. Pictures from each kidney were acquired in a double blind manner using sample numbers without the identity of the mouse.

### Statistical analyses

Data were presented as means ± standard deviation (SD) unless otherwise noted. All experiments were repeated at least three times with duplicate or triplicate samples in each assay. All data were evaluated statistically by the analysis of variance (ANOVA), followed by Nweman-Keuls multiple comparison tests using software (Prism 4.0, GraphPad Software). In the case of single mean comparison, data were analyzed by t test. P values <0.05 were regarded as statistically significant.

## Results

### Classic opiate receptors are present on podocytes

**Figure 1:**
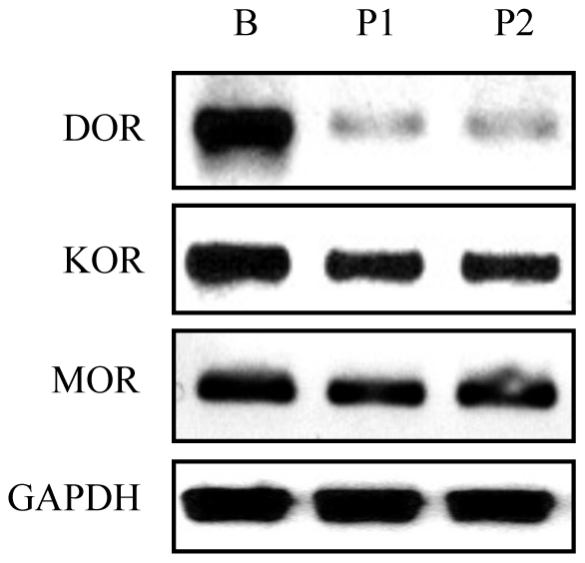
Opiate receptors are expressed in human podocytes. Total RNAs were prepared from human podocytes (labeled as P1, P2), and were used for RT-PCR to detect the expression of delta opiate receptor (DOR), kappa opiate receptor (KOR), and mu opiate receptor (MOR). RNAs from human fetal brain (B) were used as positive control. GAPDH was used as internal control.

**Figure 2:**
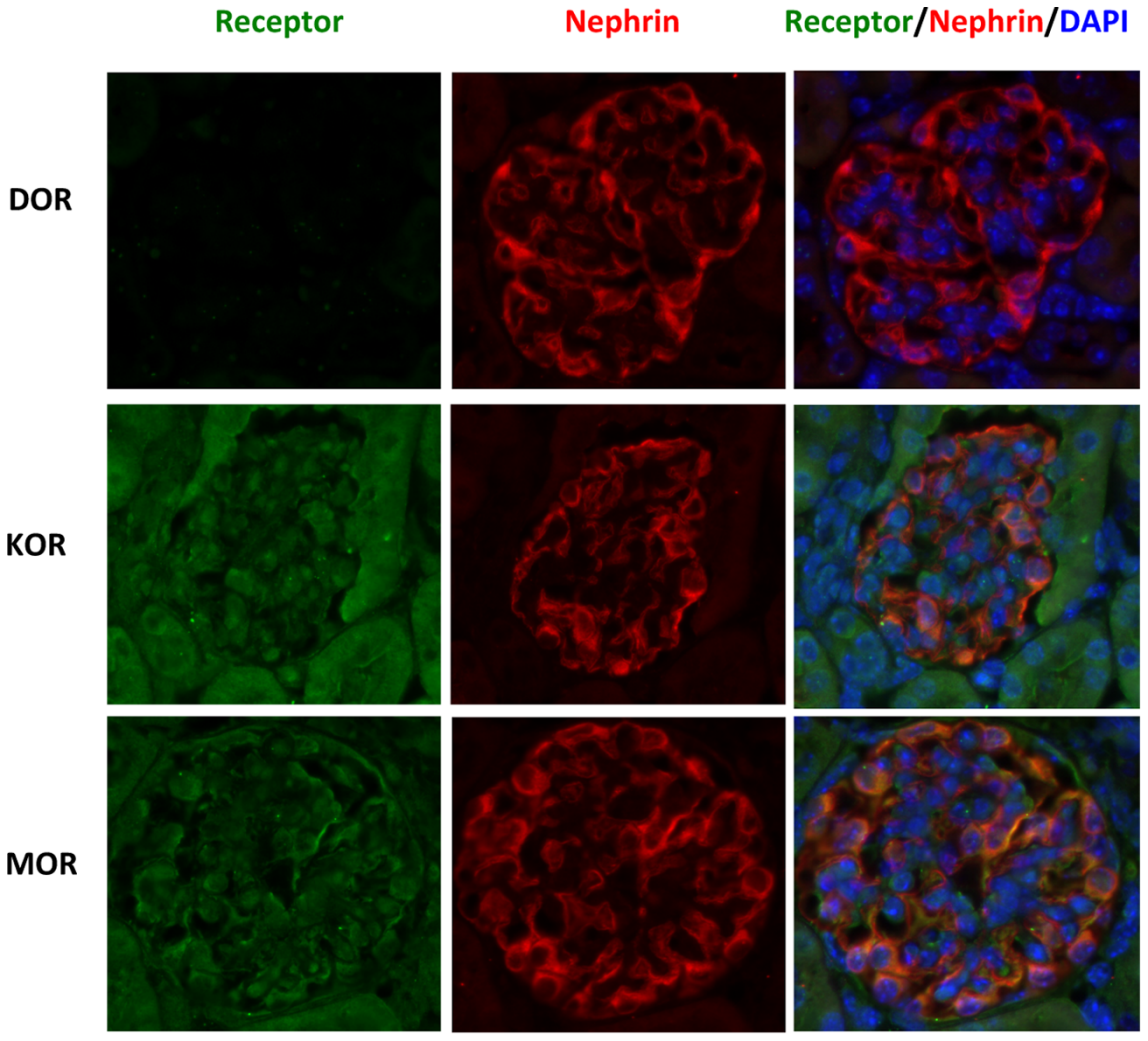
Opiate receptors are expressed in mouse podocytes. Paraffin sections were prepared from 2-month-old mice kidneys, and immunofluorescence staining was performed to detect the expression of opiate receptors. Nephrin was used as a marker of podocytes.

Opiate receptors (ORs) have been reported to express in kidney messangial cells [6], but their expression in podocytes has not been studied. Therefore, we examined the expression of ORs in podocytes. In *in vitro* study, we conducted RT-PCR analysis by using human podocytes as the RNA source. The results revealed high level expression of KOR and MOR in human podocytes, but the expression of DOR was barely detectable (Fig. 1). To confirm this observation in *in vivo* studies, we performed immunofluorescence staining of mouse kidney sections. Both KOR and MOR were highly expressed in kidney, and they were colocalized with podocyte marker nephrin; while no DOR expression was found in podocyte (Fig. 2). These results demonstrate that podocytes display expression of KOR and MOR only.

### Morphine treatment leads to acute albuminuria

**Figure 3:**
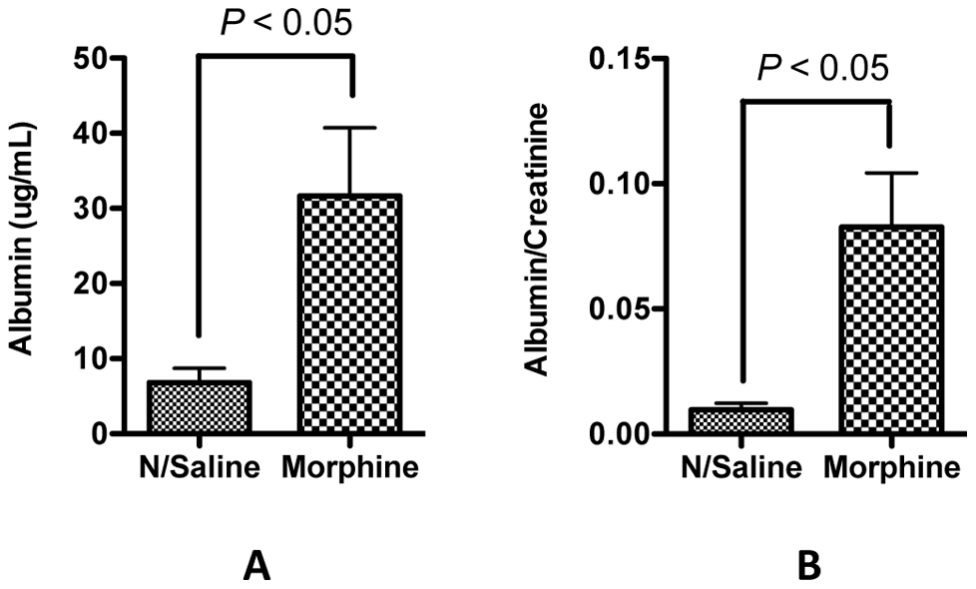
Morphine administration leads to albuminuria. Two-month old mice were administered with either normal saline or morphine by subcutaneous implantation of a 75-mg slow-release morphine pellet. After 72 h urine was collected, and the amount of albumin (**A**) and albumin/creatinine ratio (**B**) were determined.

It has been reported that chronic morphine treatment leads to glomerulophathy particularly proteinuria [5]. To examine the effect of morphine on podocytes, we treated the mice with high dose morphine as described in Materials and Methods. Three days after the treatment, mouse urine samples were collected, and total albumin and albumin/creatinine ratio were determined. The histology in Periodic acid-Schiff (PAS)-stained mouse kidney sections was also examined. Although we didn't find obvious differences between the control and treatment groups in the histology (data not shown), we did observe that high dose morphine treatment significantly increased both total albumin and albumin/creatinine ratio in the urine (Fig. 3). These results suggest that morphine treatment directly impairs the blood urine barrier.

### Morphine treatment compromises podocyte integrity

**Figure 4:**
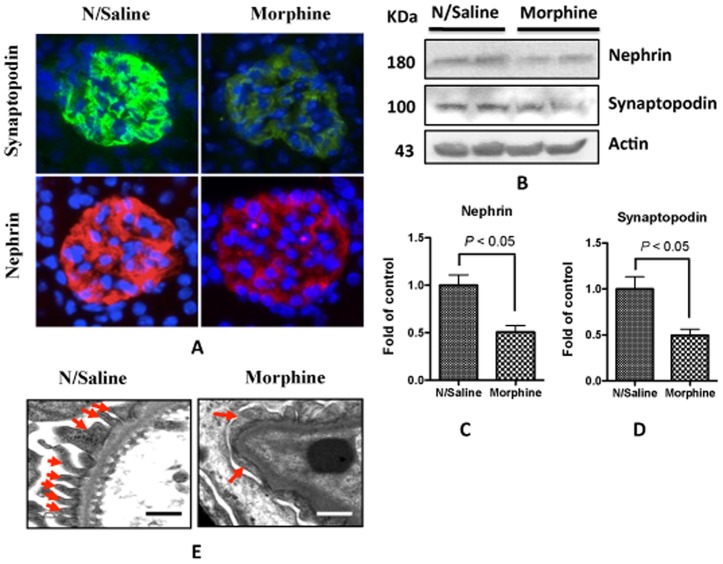
Morphine-receiving mice display alterations in SD molecules and effeacement of foot processes of podocytes. **A.** Two-month-old mice were administrated either normal saline or morphine for 3 days. Frozen sections were prepared for immuno-fluorescence staining. **B.** kidneys were homogenized, and the tissue lysates were subjected to Western blot to detect the changes of nephrin and synaptopodin. **C–D.** Quantification of the expression of nephrin (**C**) and synaptopodin (**D**), and the results (mean ± SD) represent three independent samples. **E.** A representative microphotograph from transmission electron microscopy studies. Foot processes are indicated by arrows. Foot processes are well delineated and are not fused in the control, while they are fused (effaced) in morphine-receiving mice. The scale bar is 500 nm. Mag. 270,000X.

**Figure 5:**
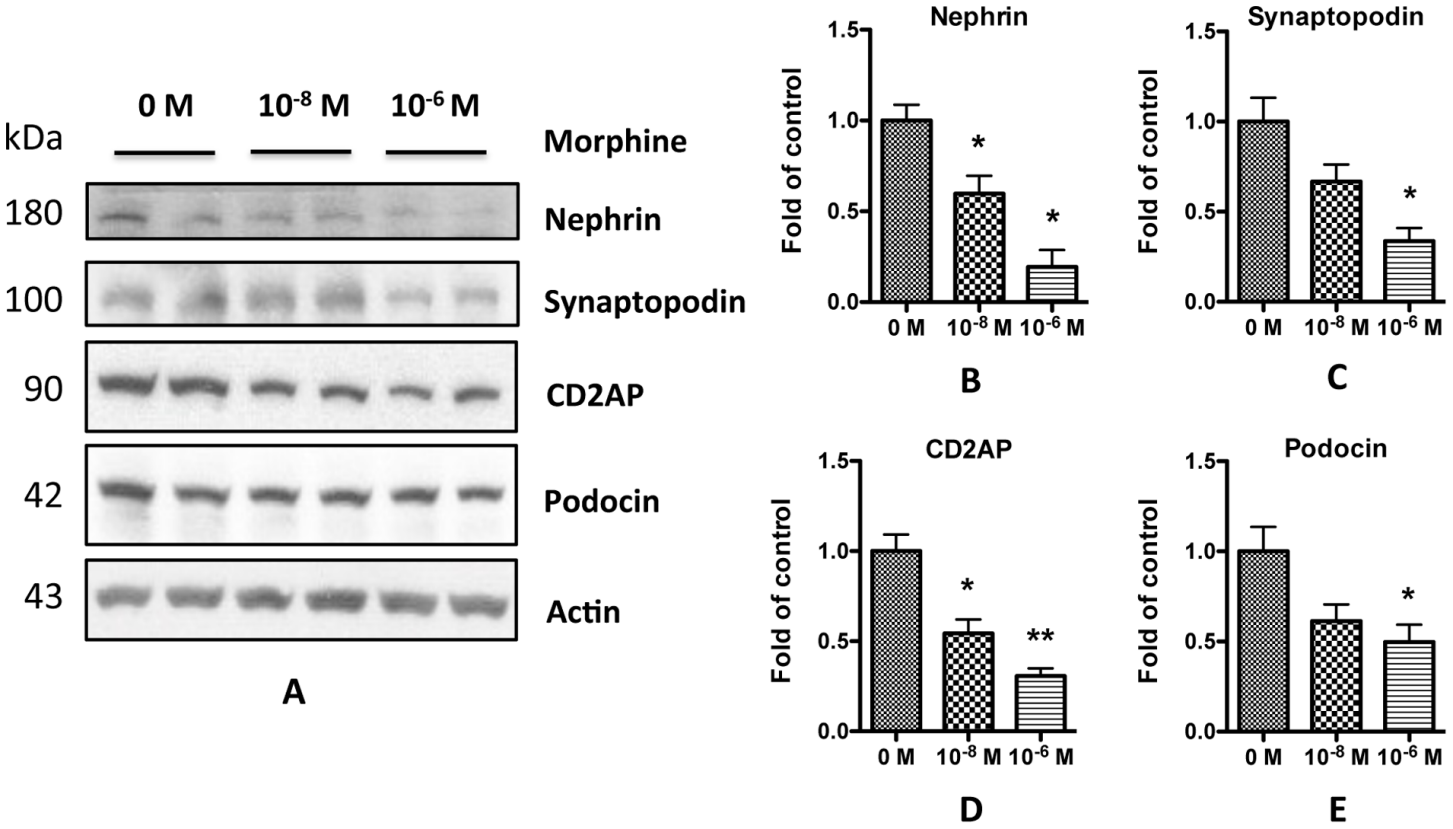
Morphine decreases podocyte expression of SD molecules. **A.** Differentiated human podocytes were treated with 10^−8^ or 10^−6^ M morphine for 24 h, and the cell lysates were collected for Western blots. **B–E.** Quantification of the expression of nephrin (**B**), synaptopodin (**C**), CD2AP (**D**), and podocin (**E**) in **A**, and the results (mean ± SD) represent three independent samples. * p<0.05 compared with control (0 M).

To determine the effect of morphine on podocytes, we performed immunofluorescent staining of mouse kidney sections and immunoblotting studies of the kidney lysates. Morphine attenuated the expression of podocyte specific molecules such as synaptopodin and nephrin (Fig. 4, A–D). *In vitro* study also showed that morphine treatments decreased the protein expression of SDCMs in human podocytes (Fig. 5). Transmission electron microscopy of the mouse kidney sections showed that morphine administration increased the podocyte foot process effacement (Figure 4E). These results indicate that morphine treatment may compromise podocyte integrity.

### Morphine mediates alterations in podocyte SDCMs via MOR and KOR

**Figure 6:**
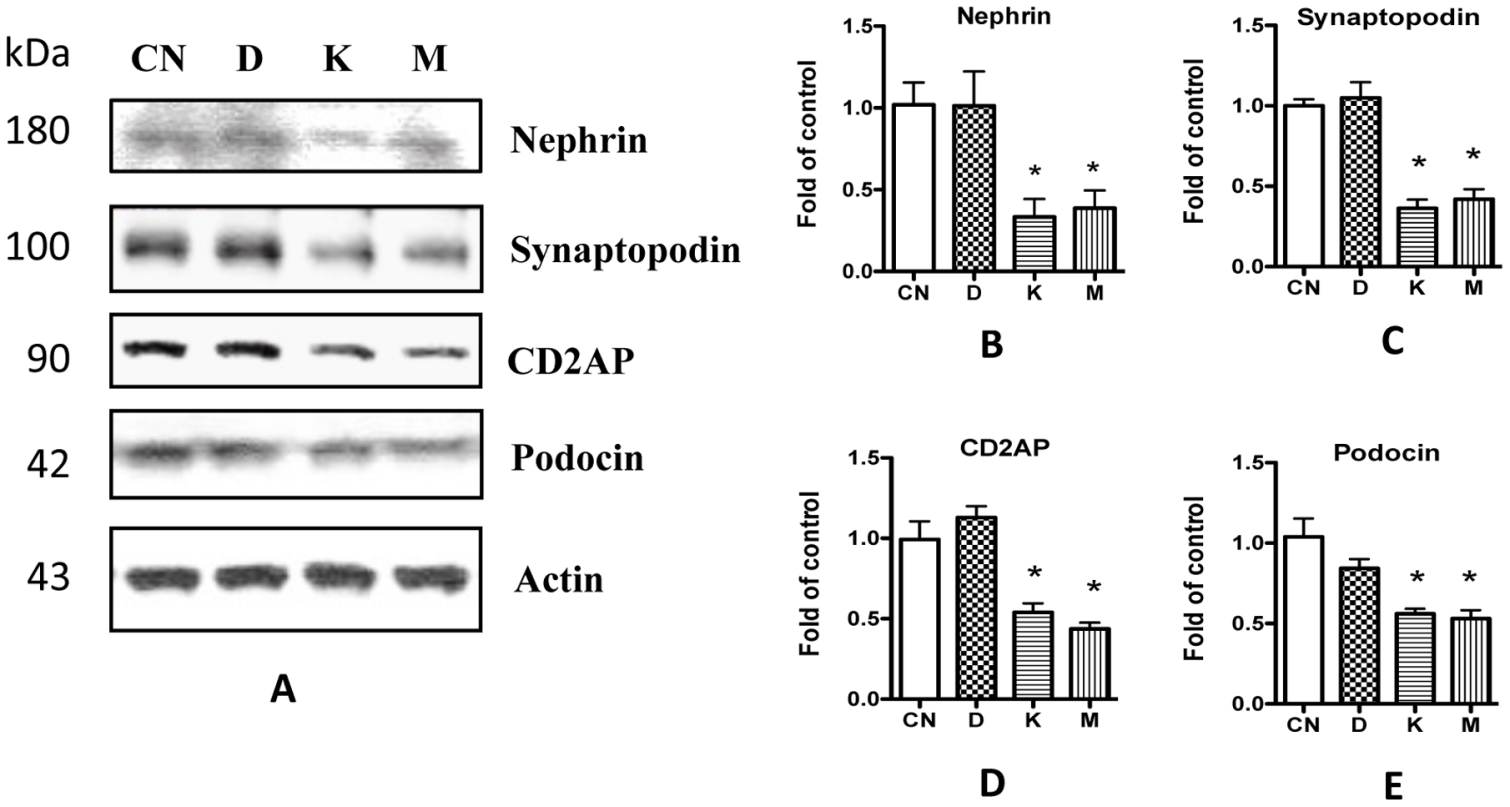
Morphine affects podocytes through K and M receptors. **A.** Human podocytes were treated with agonists specifically for DOR (labeled as D), KOR (K), or MOR (M) for 24 h, and the cell lysates were collected for Western blots. **B–E.** Quantification of the expression of nephrin (**B**), synaptopodin (**C**), CD2AP (**D**), and podocin (**E**) in **A**, and the results (mean ± SD) represent three independent samples. * p<0.05 compared with control (CN).

Weber *et al* reported that morphine affects mesangial cells mainly via KOR but not MOR [6]. To determine the receptors involved in podocytes, we stimulated human podocytes with OR-specific agonists. Western blot results revealed that both KOR-specific agonist LKD26 and MOR-specific agonist DAMGO decreased the expression of SDCMs, while DOR-specific agonist DPDPE did not show this function (Fig. 6). These results indicate that morphine-induced alterations in podocyte SDCM occurred via both KOR and MOR.

### Morphine induces podocyte DNA damage through ROS generation

**Figure 7:**
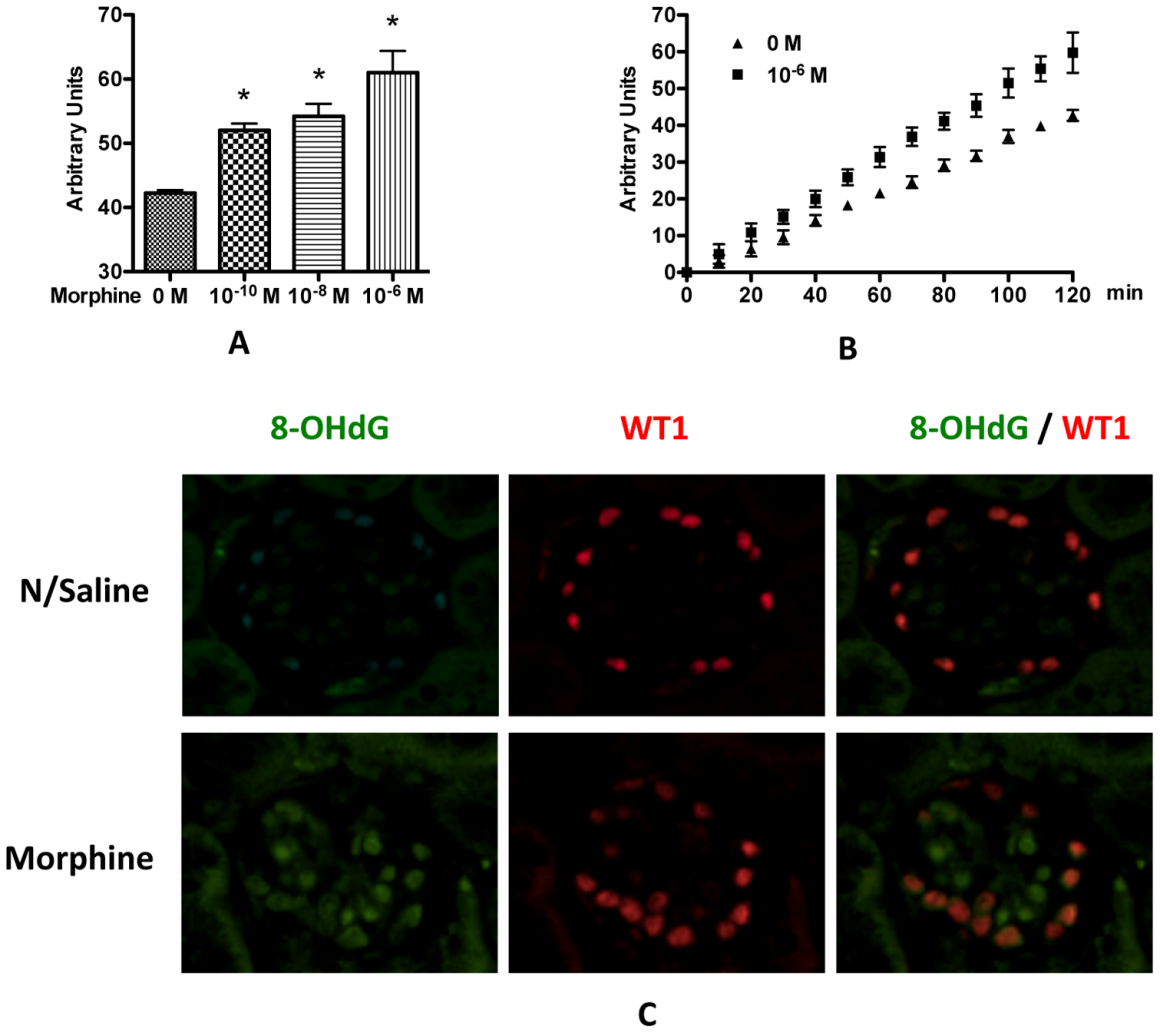
Morphine increases ROS generation in podocyte. **A.** Human podocytes were treated with 10^−10^ to 10^−6^ M morphine for 12 h, and were labeled with DCFH for 30 min. After washing with PBS, the cells were incubated at room temperature for 2 h, and the ROS generation was determined. * p<0.001 compared with control (0 M). **B.** Kinetics of ROS generation for 10^−6^ M morphine treatment. **C.** Paraffin sections were prepared from control or morphine-receiving mice, and immunofluorescent staining was performed to detect the nuclear expression of 8-OHdG, a molecular marker of oxidative damage to DNA. WT1 was used as a marker of podocytes.

Our group and others have demonstrated that ROS is a significant contributing factor for podocyte injury and for the progression of chronic kidney disease [8], [19], [20]. ROS has also been shown to damage SD [21], [22], [23]. To examine the effect of morphine on intracellular ROS production, the fluorescence intensity of the intracellular fluoroprobe (DCFH) was evaluated. Results showed that ROS generation was significantly increased in morphine-treated cells as compared with unstimulated cells (Figure 7). Morphine increased ROS generation in a dose-dependent manner (Figure 7A).

To determine the effect of morphine-induced ROS generation on oxidative DNA damage in the podocyte, we carried out immunofluorescent staining of podocytes for 8-hydroxyguanine (8-OHdG), a molecular marker of oxidative damage to DNA, in morphine-receiving mice. Morphine-receiving mice displayed increased positive staining of 8-OHdG in podocytes (Figure 7C).

**Figure 8:**
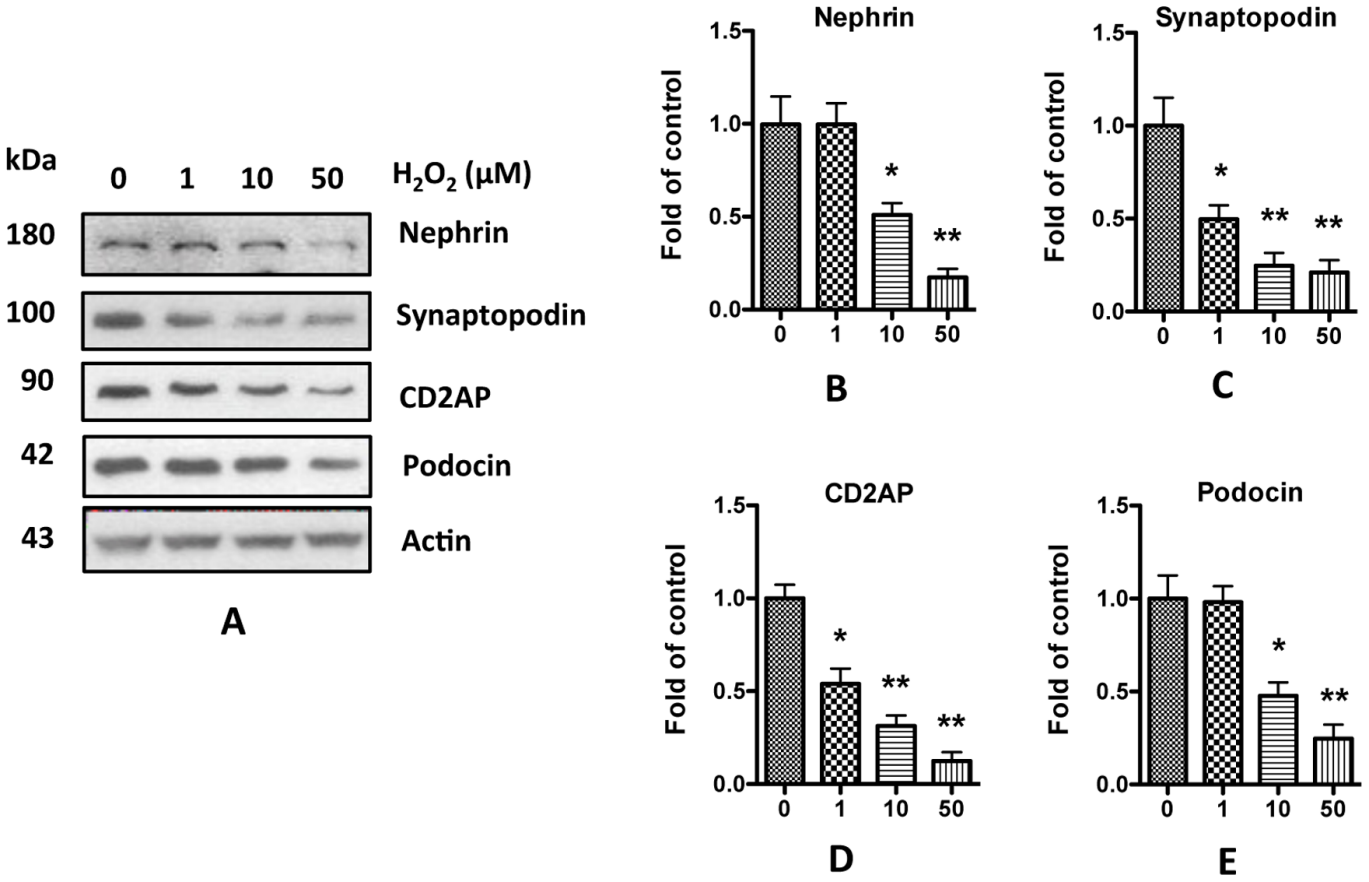
H_2_O_2_ decreases podocyte expression of SD molecules. **A.** Human podocytes were treated with 1, 10, or 50 µM H_2_O_2_ for 24 h, and the cell lysates were collected for Western blots. **B–E.** Quantification of the expression of nephrin (**B**), synaptopodin (**C**), CD2AP (**D**), and podocin (**E**) in **A**, and the results (mean ± SD) represent three independent samples. * p<0.05, ** p<0.01 compared with control (0 µM).

**Figure 9:**
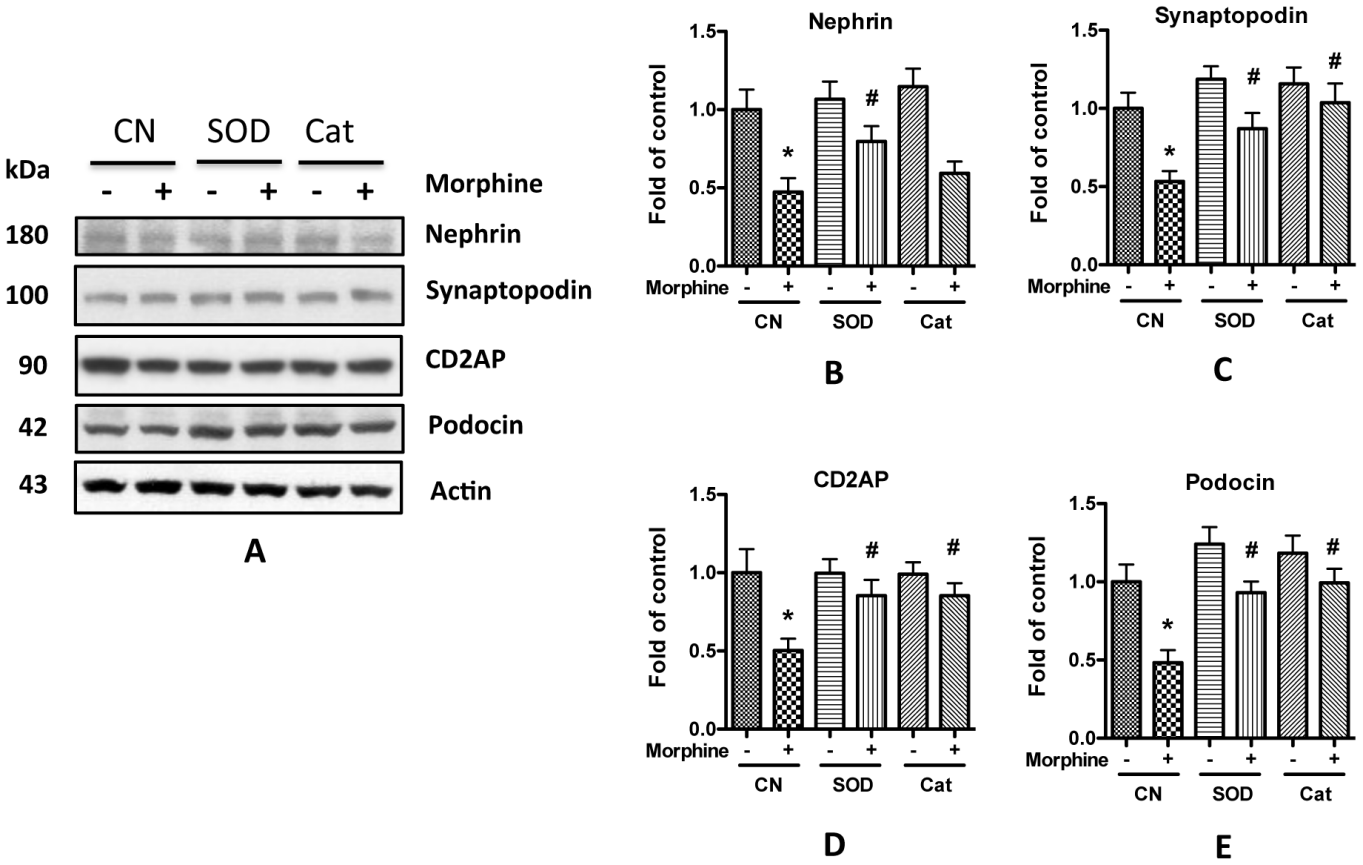
SOD and catalase attenuate morphine-induced decrease of podcoyte expression of SD molecules. **A.** Human podocytes were pretreated with SOD or catalase for 1 h before 10^−6^ M morphine was added. After another 24 h incubation at 37°C, the cell lysates were collected for Western blot. **B–E.** Quantification of the expression of nephrin (**B**), synaptopodin (**C**), CD2AP (**D**), and podocin (**E**) in **A**, and the results (mean ± SD) represent three independent samples. * p<0.05 compared with blank control, while # p<0.05 compared with morphine treatment alone.

To test the effect of oxidative stress on SDCMs, aliquots of variable concentrations of hydrogen peroxide (H_2_O_2_) were added to the media of human podocytes for 24 h. Western blotting studies revealed that H_2_O_2_ decreased SDCMs expression in a dose-dependent manner (Figure 8). To further confirm these observations, podocytes were pre-treated with either superoxide dismutase or catalase and then incubated in media containing H_2_O_2_. As shown in Figure 9, pretreating the human podocytes with SOD or catalase significantly attenuated morphine-induced compromise of SDCMs. Taken together, these data suggest that morphine-induced ROS generation may be a contributor to the decrease of SDCMs.

### AKT, JNK, and p38 pathways are involved in opioid-induced podocyte injury

**Figure 10:**
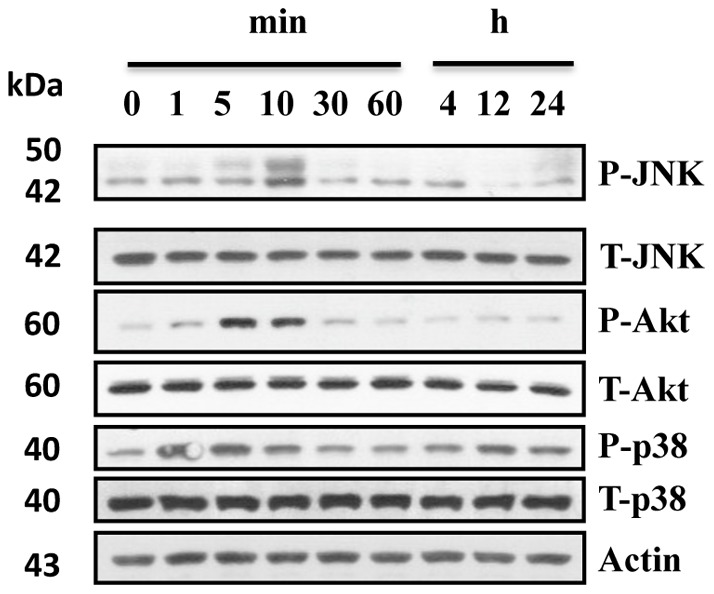
Morphine activates AKT, p38 and JNK. Human podocytes were starved in serum free medium for 12 h, and then 10^−6^ M morphine was added. Cell lysates were collected at different time points for Western blot.

Several kinases, including AKT, JNK, Erk1/2 and p38 have been implicated in podocyte injury and the progression of chronic kidney diseases (CKD) [6], [15], [24], [25], [26]. A transcription factor, signal transducer and activator of transcription 3 (STAT3), has also been reported to regulate HIV-induced podocyte proliferation [24]. To examine the involvement of these kinases and factors in morphine-induced down-regulation of SDCMs, we first evaluated the phosphorylation of these proteins. We treated the human podocytes with morphine, and collected the cell lysates at different time points for Western blot studies. Results showed that morphine stimulation significantly activated AKT, JNK, and p38 at early time points (Fig. 10), while neither Erk1/2 nor STAT3 could be activated by morphine at any time point (data not shown). We also used KOR- and MOR-specific agonists, LKD26 and DAMGO, to stimulate the human podocytes, and the same activation results with morphine were observed (data not shown). These results suggest that morphine down-regulates SDCMs via AKT, JNK, and p38, but not Erk1/2 or STAT3.

**Figure 11:**
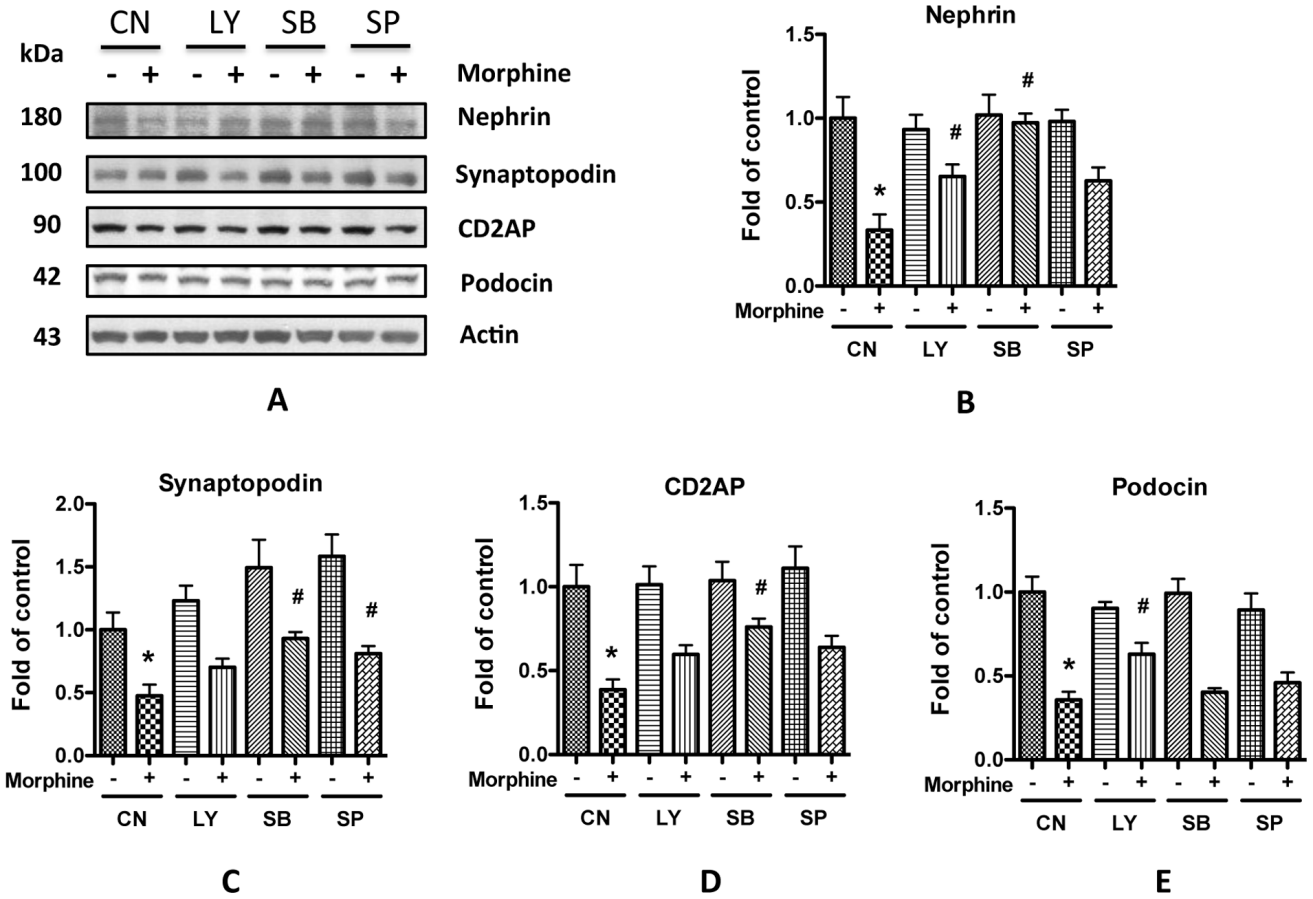
Inhibition of AKT, p38, and JNK attenuates morphine-induced decrease of SD molecules expression. **A.** Human podocytes were pretreated with of LY294002 (LY), SB203580 (SB), or SP600125 (SP) for 1 h before 10^−6^ M morphine was added. After another 24 h incubation at 37°C, the cell lysate were collected for Western blot. **B–E.** Quantification of the expression of nephrin (**B**), synaptopodin (**C**), CD2AP (**D**), and podocin (**E**) in **A**, and the results (mean ± SD) represent three independent samples. * p<0.05 compared with blank control, while # p<0.05 compared with morphine treatment alone.

To further examine the role of activation of AKT, JNK, and p38 in morphine-induced down-regulation of SDCMs, the expression of SDCMs was measured in podocytes treated with morphine in the presence or absence of LY294002, an inhibitor of PI13/AKT, SP600125, an inhibitor of JNK, or SB203580, an inhibitor of p38. As presented in Figure 11, all these inhibitors partially attenuated morphine-induced down-regulation of SDCMs. These results indicate that AKT, JNK, and p38 pathways are involved in the regulation of morphine-induced impairment of SDCMs.

## Discussion

Morphine has been reported to cause kidney cell injury in both *in vitro* and *in vivo* studies [7], [8], [9], [10], [11], [12]. In the current study, we observed that administration of morphine contributed to albuminuria which appeared to be glomerular in origin and a consequence of the loss of slit diaphragm integrity [27]. Both *in vivo* and *in vitro* data confirmed that morphine inflicted podocyte injury in the form of attenuated expression of SDCMs (Figure 4 and 5); these findings further confirmed that loss of integrity of SD contributed to albuminuria in morphine-receiving mice. Since these SDCMs are actin-associated molecules, the reduced level of expression of these molecules by morphine may change the actin cytoskeleton organization. Transmission electron microscopy of the mouse kidney sections showed effacement of podocyte foot process in morphine-receiving mice (Figure 4E), which might be due to the change of the actin cytoskeleton organization. However, further studies are needed to explore this aspect.

In previously reported studies, morphine has been demonstrated to exert bimodal effects (both apoptosis and proliferation) on glomerular epithelial cells [8]; however the role of opiate receptors was not evaluated in these studies. In the current study, we found that KOR and MOR, but not DOR, were expressed in both human and mouse podocytes (Figures 1 and 2). We also found that stimulating either KOR or MOR in podocytes could activate related kinases and down-regulate SDCMs. To our knowledge, this is the first report on the opiate receptors in podocytes. Weber *et al* reported that although both KOR and MOR were expressed in glomerular mesangial cells, only KOR played the function to activate STAT3 and led to the proliferation and glomerulopathy [6]. These findings indicate that podocytes behave differently from mesangial cells in morphine milieu.

Oxidative stress is a common cause of cellular injury. Several investigators have demonstrated that enhanced ROS generation leads to albuminuria by damaging SD components [21], [28], [29]. Morphine has been demonstrated to stimulate the production of superoxide by macrophage and mesangial cells [22], [23]. As we previously reported, generation of ROS induces apoptosis of rat glomerular epithelial cells [8]. Here we also found that morphine increased ROS generation in human podocytes in a dose-dependent manner (Figure 7A). Addition of H_2_O_2_ to the media decreased the expression of SDCMs (Figure 8), while free radical scavengers prevented this damaging effect of morphine (Figure 9). All these findings strongly suggest the role of ROS in morphine-induced down-regulation of SDCMs. One potential possibility is that ROS induced injury hampers the expression of SDCMs. Further investigation into the detailed underlying mechanisms need to be carried out in future studies.

Investigating the kinase or transcription factor pathways involved in morphine-induced kidney injury may provide insight into new potential targets for therapy. STAT3, AKT and mitogen-activated protein (MAP) kinases, including ERK1/2, JNK, and p38, have been implicated in podocyte injury and the progression of chronic kidney diseases (CKD) [6], [14], [24], [25], [26]. All these kinases or transcription factors may also be activated by morphine in various cells [6], [30], [31]. We examined the effect of morphine on the activation of these kinases and factors in podocytes. Our results revealed that morphine stimulated the phosphorylation of AKT, JNK and p38 (Figure 10), but could not activate Erk1/2 or STAT3 (data not shown). These observations were further confirmed by the use of KOR and MOR specific agonists on human podocytes in morphine milieu (data not shown).

Takano *et al* reported that activation of AKT suppressed the expression of nephrin [32]; Ikezumi *et al* claimed that activation of JNK or p38 decreased the expression of nephrin and podocin, while inhibiting these kinases restored their expressions [33]. Consistent with these reports, we also found that inhibiting AKT, JNK, and p38 could partially prevent the morphine-induced decrease of SDCMs (Figure 11), indicating the regulation of these three kinases in morphine-induced SD damage.

In conclusion, we have demonstrated that morphine has the potential to directly impair the SDCMs in podocytes, which will contribute to acute kidney injury. The effects of morphine on podocytes are mediated through both MOR and KOR. These impairments are through generation of ROS, and are regulated by AKT, JNK, and p38 pathways. Our study provides insight into new mechanisms involved in morphine-induced podocyte damage, and highlights some new therapeutic targets for morphine induced kidney injury.

## References

[1] MacPherson RD (2000) The pharmacological basis of contemporary pain management. Pharmacol Ther 88: 163–185. 11150596 10.1016/s0163-7258(00)00090-5

[2] Dasgupta N, Kramer ED, Zalman MA, Carino S Jr, Smith MY, et al (2006) Association between non-medical and prescriptive usage of opioids. Drug Alcohol Depend 82: 135–142. 16236466 10.1016/j.drugalcdep.2005.08.019

[3] Hopfer CJ, Mikulich SK, Crowley TJ (2000) Heroin use among adolescents in treatment for substance use disorders. J Am Acad Child Adolesc Psychiatry 39: 1316–1323. 11026188 10.1097/00004583-200010000-00021

[4] Nielsen DA, Kreek MJ (2012) Common and specific liability to addiction: Approaches to association studies of opioid addiction. Drug Alcohol Depend Suppl 1: S33–41. 10.1016/j.drugalcdep.2012.03.026 PMC3689423 22542464

[5] Paulozzi LJ, Budnitz DS, Xi Y (2006) Increasing deaths from opioid analgesics in the United States. Pharmacoepidemiol Drug Saf 15: 618–627. 16862602 10.1002/pds.1276

[6] Weber ML, Farooqui M, Nguyen J, Ansonoff M, Pintar JE, et al (2008) Morphine induces mesangial cell proliferation and glomerulopathy via kappa-opioid receptors. Am J Physiol Renal Physiol 294: 1388–1397. 10.1152/ajprenal.00389.2007 18385270

[7] Mongia A, Bhaskaran M, Reddy K, Manjappa N, Baqi N, et al (2004) Protease inhibitors modulate apoptosis in mesangial cells derived from a mouse model of HIVAN. Kidney Int 65: 860–870. 14871405 10.1111/j.1523-1755.2004.00464.x

[8] Patel J, Manjappa N, Bhat R, Mehrotra P, Bhaskaran M, et al (2003) Role of oxidative stress and heme oxygenase activity in morphine-induced glomerular epithelial cell growth. Am J Physiol Renal Physiol 285: F861–869. 12812915 10.1152/ajprenal.00134.2003

[9] Singhal PC, Sagar S, Reddy K, Sharma P, Ranjan R, et al (1998) HIV-1 gp120 envelope protein and morphine-tubular cell interaction products modulate kidney fibroblast proliferation. J Investig Med 46: 243–248. 9676058

[10] Singhal PC, Sharma P, Sanwal V, Prasad A, Kapasi A, et al (1998) Morphine modulates proliferation of kidney fibroblasts. Kidney Int 53: 350–357. 9461094 10.1046/j.1523-1755.1998.00758.x

[11] Bourgoignie JJ (1990) Renal complications of human immunodeficiency virus type 1. Kidney Int 37: 1571–1584. 2194069 10.1038/ki.1990.151

[12] Rao TKS, Filppone EJ, Nicastri AD, Landesman H, Frank E, et al (1984) Associated focal segmental glomerulosclerosis in the acquired immunodeficiency syndrome. N Engl J Med 310: 664–673. 10.1056/NEJM198403153101101 6700641

[13] Johnson JE Jr, White JJ Jr, Walovitch RC, London ED (1987) Effects of morphine on rat kidney glomerular podocytes: a scanning electron microscopic study. Drug Alcohol Depend 19: 249–257. 3595448 10.1016/0376-8716(87)90044-5

[14] Benzing T (2004) Signaling at the slit diaphragm. J Am Soc Nephrol 15: 1382–1391. 15153549 10.1097/01.asn.0000130167.30769.55

[15] Mao J, Zhang Y, Du L, Dai Y, Yang C, et al (2006) Expression profile of nephrin, podocin, and CD2AP in Chinese children with MCNS and IgA nephropathy. Pediatr Nephrol 21: 1666–1675. 16941146 10.1007/s00467-006-0218-z

[16] Xing Y, Ding J, Fan Q, Guan N (2006) Diversities of podocyte molecular changes induced by different antiproteinuria drugs. Exp Biol Med (Maywood) 231: 585–593. 16636307 10.1177/153537020623100513

[17] Husain M, Meggs LG, Vashistha H, Simoes S, Griffiths KO, et al (2009) Inhibition of p66ShcA longevity gene rescues podocytes from HIV-1-induced oxidative stress and apoptosis. J Biol Chem 284: 16648–16658. 19383602 10.1074/jbc.M109.008482 PMC2713565

[18] Salhan D, Sagar A, Kumar D, Rattanavich R, Rai P, et al (2012) HIV-associated nephropathy: role of AT2R. Cell Signal 24: 734–741. 22108089 10.1016/j.cellsig.2011.11.007 PMC3258382

[19] Nistala R, Whaley-Connell A, Sowers JR (2008) Redox control of renal function and hypertension. Antioxid Redox Signal 10: 2047–2089. 18821850 10.1089/ars.2008.2034 PMC2582196

[20] Susztak k, Raff AC, Schiffer M, Böttinger EP (2006) Glucose-induced reactive oxygen species cause apoptosis of podocytes and podocyte depletion at the onset of diabetic nephropathy. Diabetes 55: 225– 233. 16380497

[21] Marshall SM (2007) The podocyte: a potential therapeutic target in diabetic nephropathy? Curr Pharm Des 13: 2713–2720. 17897015 10.2174/138161207781662957

[22] Sharp BM, Keane WF, Suh HJ, Gekker G, Tsukayama D, et al (1985) Opioid peptides rapidly stimulate superoxide production by human polymorphonuclear leukocytes and macrophages. Endocrinology 117: 793–795. 2862014 10.1210/endo-117-2-793

[23] Singhal PC, Pamarthi M, Shah R, Chandra D, Gibbons N (1994) Morphine stimulates superoxide formation by glomerular mesangial cells. Inflammation 18: 293–299. 8088925 10.1007/BF01534270

[24] Husain M, Sunamoto M, D'Agati VD, Klotman ME, Iyengar R, et al (2004) Nef stimulates proliferation of glomerular podocytes through activation of Src-dependent Stat3 and MAPK1, 2 pathways. J Clin Invest 114: 643–651. 15343382 10.1172/JCI21004 PMC514582

[25] Koshikawa M, Mukoyama M, Mori K, Suganami T, Sawai K, et al (2005) Role of p38 mitogen-activated protein kinase activation in podocyte injury and proteinuria in experimental nephrotic syndrome. J Am Soc Nephrol 16: 2690–2701. 15987752 10.1681/ASN.2004121084

[26] Lim AK, Nikolic-Paterson DJ, Ma FY, Ozols E, Young MJ, et al (2009) Role of MKK3-p38 MAPK signalling in the development of type 2 diabetes and renal injury in obese db/db mice. Diabetologia 52: 347–358. 19066844 10.1007/s00125-008-1215-5

[27] Huber TB, Benzing T (2005) The slit diaphragm: a signaling platform to regulate podocyte function. Curr Opin Nephrol Hypertens 14: 211–216. 15821412 10.1097/01.mnh.0000165885.85803.a8

[28] Dzau VJ (2001) Theodore Cooper Lecture: Tissue angiotensin and pathology of vascular disease: A unifying hypothesis. Hypertension 37: 1047–1052. 11304501 10.1161/01.hyp.37.4.1047

[29] Tojo A, Asaba K, Onozato ML (2007) Suppressing renal NADPH oxidase to treat diabetic nephropathy. Expert Opin Ther Targets 11: 1011–1018. 17665974 10.1517/14728222.11.8.1011

[30] Ma W, Zheng WH, Powell K, Jhamandas K, Quirion R (2001) Chronic morphine exposure increases the phosphorylation of MAP kinases and the transcription factor CREB in dorsal root ganglion neurons: an in vitro and in vivo study. Eur J Neurosci 14: 1091–1104. 11683901 10.1046/j.0953-816x.2001.01731.x

[31] Wen H, Lu Y, Yao H, Buch S (2011) induces expression of platelet-derived growth factor in human brain microvascular endothelial cells: implication for vascular permeability. PLoS One 6: e21707. 21738771 10.1371/journal.pone.0021707 PMC3125302

[32] Takano Y, Yamauchi K, Hayakawa K, Hiramatsu N, Kasai A, et al (2007) Transcriptional suppression of nephrin in podocytes by macrophages: roles of inflammatory cytokines and involvement of the PI3K/Akt pathway. FEBS Lett 581: 421–426. 17239861 10.1016/j.febslet.2006.12.051

[33] Ikezumi Y, Suzuki T, Karasawa T, Kawachi H, Nikolic-Paterson DJ, et al (2008) Activated macrophages down-regulate podocyte nephrin and podocin expression via stress-activated protein kinases. Biochem Biophys Res Commun 376: 706–711. 18809387 10.1016/j.bbrc.2008.09.049

